# The Relationship Between Therapeutic Alliance, Early Symptom Change, and Outcome Among In‐Patients With Anorexia Nervosa

**DOI:** 10.1002/eat.24524

**Published:** 2025-09-24

**Authors:** Janina Werz, Brunna Tuschen‐Caffier, Ulrich Voderholzer

**Affiliations:** ^1^ Department of Clinical Psychology Albert‐Ludwigs‐University Freiburg Freiburg Germany; ^2^ Department of Clinical Psychology and Trauma Therapy University of the Bundeswehr Munich Neubiberg Germany; ^3^ Schön Klinik Roseneck Prien Am Chiemsee Germany; ^4^ Department of Psychiatry and Psychotherapy University Hospital, Ludwig Maximilians University Munich Germany; ^5^ Department of Psychiatry and Psychotherapy Albert‐Ludwigs‐University Freiburg Freiburg Germany

**Keywords:** anorexia nervosa, early change, eating disorder, outcome, premature termination, therapeutic alliance, working alliance

## Abstract

**Objective:**

Therapeutic alliance (TA) is an important process variable, evidence suggesting its influence on treatment outcome across several mental disorders. Yet, its role in treating anorexia nervosa (AN) remains under‐examined. Previous research has yielded heterogeneous results, indicating a complex relationship between TA, early change, and outcome, potentially depending on contributing factors like age. This relationship was examined in a sample of 173 adult and adolescent inpatients with AN treated in a specialized clinic.

**Method:**

Data were collected in weeks 2, 5, 9, and the week before discharge. Outcome was defined dimensionally (Eating Disorder Examination Questionnaire, Body Mass Index) and categorically (premature termination, weight status, remission status). Several potential influencing factors (e.g., duration of illness, previous therapy) were analyzed. The effect of TA on early change and outcome, and vice versa, was analyzed using regression analyses.

**Results:**

TA was positively associated with greater early weight gain and overall general symptom change. Later TA predicted premature termination and remission status. The latter effect ceased when early change was considered. The TA subscale “task” played the most important role. Early change in ED symptoms predicted better TA later in treatment.

**Discussion:**

In accordance with previous research, a complex bidirectional relationship between TA and outcome in AN patients was identified. The results indicate the importance of focusing on symptom improvement as well as establishing an agreement on therapeutic tasks. Future research should concentrate on the complexity and bidirectionality of TA and symptom change over the course of treatment, considering possible mediators.

**Summary**

- Treatment success rates for anorexia nervosa are still not high enough and could be enhanced using factors that are important during treatment.
- Therapeutic alliance plays a small but significant role in facilitating a better outcome for patients with anorexia nervosa and in preventing them from prematurely terminating treatment.
- The agreement between therapist and patient on the therapeutic task seems to be most important for this effect.
- Enabling patients to quickly gain weight and change their eating behaviors early on is an important predictor of treatment success and later therapeutic alliance.

## 1 Introduction

Eating disorders (ED) are severe and complex mental disorders that pose a significant burden on those affected and their social environment. While several manualized, validated, disorder‐specific as well as transdiagnostic approaches and guidelines (Crone et al. 2023; National Guideline Alliance (UK) 2017; Resmark et al. 2019; Shapiro et al. 2007; Svaldi et al. 2019; Watson and Bulik 2013) exist for the treatment of ED, remission rates are still unsatisfactory. Approximately one third of patients with anorexia nervosa (AN) and bulimia nervosa (BN) experience a chronic course of illness, with only 30%–50% achieving remission even after treatment in a specialized ED setting (Brockmeyer et al. 2018; Schlegl et al. 2015; Solmi et al. 2024; Svaldi et al. 2019). Given the numerous severe adverse effects of AN on emotional, cognitive, physical, and social well‐being and functioning, alongside highly elevated mortality and morbidity rates (Arcelus et al. 2011; Fichter and Quadflieg 2016; Quadflieg et al. 2019; Solmi et al. 2024), there is an urgent need to improve treatment outcomes.

Several studies have investigated predictors of ED outcome, examining baseline characteristics like symptom severity, duration of illness, and comorbidities (for a recent review, see Gorrell et al. 2023). These variables can help enhance our understanding of which patient group may benefit from adaptations in therapeutic strategies and should be regarded as important influencing variables in outcome studies.

Another aspect to consider is the role of process‐related variables, which have been highlighted by international guidelines and ED literature as significant factors for successful treatment (Brauhardt et al. 2014; Crone et al. 2023; Herpertz et al. 2019; National Guideline Alliance (UK) 2017). One of those variables is the therapeutic alliance (TA). While there are different conceptualizations of this latent construct, most research is based on Bordin's (1979) pantheoretical model, consisting of three elements of TA: agreement on the goals of treatment, agreement on the tasks necessary to be accomplished, and the quality of the bond between patient and therapist. Although TA can be measured through observer ratings, it is most commonly assessed using self‐report questionnaires.

While a substantial body of literature demonstrates the positive impact of TA on treatment outcome and dropout rates for a wide range of disorders across various treatment types and contexts (Del Re et al. 2021; Flückiger et al. 2018, 2020; Iovoli et al. 2024), there are heterogeneous results for its impact on ED patients (Flückiger et al. 2018). Recent reviews and meta‐analyses have concluded that the effect of TA on outcome for ED treatments may vary across samples, especially between diagnoses (AN vs. BN) and age groups (adults vs. adolescents) (Antoniou and Cooper 2013; Graves et al. 2017; Werz et al. 2022; Zaitsoff et al. 2015). However, the quality of the included studies has often been limited, with most studies investigating TA in secondary analyses, resulting in insufficient power, lack of consideration for confounding variables, and small sample sizes. Furthermore, most studies have focused on homogeneous samples regarding diagnoses and age group, restricting comparisons of effects between those groups. Gorrell et al. (2023) concluded their review on (baseline) predictor variables for ED outcome by recommending not only the inclusion of these variables in outcome studies but the examination of heterogeneous samples treated in ecologically valid settings as opposed to university study centers.

Moreover, the dynamic interplay between symptom improvement and TA is not yet fully understood. Several studies suggest that the relationship is bidirectional, with a positive impact of early symptom change on later TA (Flückiger et al. 2020; Graves et al. 2017). Hence, it remains unclear which of these variables serves as the primary driver of a successful outcome. The question of whether TA or early change poses as the more relevant factor is a relevant question, especially in the treatment of AN, where an early and intense focus on reducing symptoms remains critically debated in the therapeutic community. Due to factors like high ego‐syntonicity, fear of loss of control, and a high ambivalence regarding the motivation to change, a strict and directive, symptom‐focused therapeutic stance is discussed as a factor that may lead to a decrease in TA, worse clinical outcomes, and a rise in premature treatment termination (Brown et al. 2013b; Robertson and Thornton 2021; Vinchenzo et al. 2022).

### 1.1 Aim

Against this background, this study aims to enhance the understanding of the complex relationship between outcome and TA among in‐patients with AN. The study employs a theory‐based and hypotheses‐driven approach, building on the previous research on TA and early change (Del Re et al. 2021; Flückiger et al. 2018, 2020; Graves et al. 2017; Iovoli et al. 2024; Rienecke et al. 2016; Sly et al. 2013; Werz et al. 2022). To establish the comparability of different age groups, we used a sample in which these different groups were treated within a single naturalistic setting, examining the following hypotheses.

### 1.2 Hypotheses

1. High TA at week 2, week 5, and week 9 of treatment is associated with a higher improvement of ED symptomatology during treatment (from admission to week 5/week before discharge).
2. High TA at week 2, week 5, and week 9 of treatment is associated with a lower probability of treatment drop out.
3. A higher improvement of ED symptomatology after 4 weeks (early change) of treatment is associated with a higher TA at weeks 5, 9, and at discharge.
4. After controlling for the effect of early change in ED symptomatology, the effects described in hypotheses 1 and 2 will still occur.
5. The effect of TA on treatment outcome varies depending on the age group of patients, with a higher effect for adolescents and patients with AN.

Additionally, the differential effect of the WAI subscales (goals, tasks, bond) was investigated in an exploratory manner.

## 2 Method

### 2.1 Participants

To be included, patients had to be seeking in‐patient treatment for an ED at the study center (Schoen Clinic Roseneck), be at least 14 years old, and have their diagnosis of AN or BN confirmed through a clinical interview. Due to a small number of BN patients, which led to an underpowered subsample, BN patients were post hoc excluded from all analyses reported. All patients included were treated in wards with the same therapeutic concepts and the same number of individual sessions (2/week). Exclusion criteria were conditions preventing treatment at the study center—i.e., current psychosis, acute suicidality, substance dependencies, and court order for treatment.

### 2.2 Procedure

**FIGURE 1:**
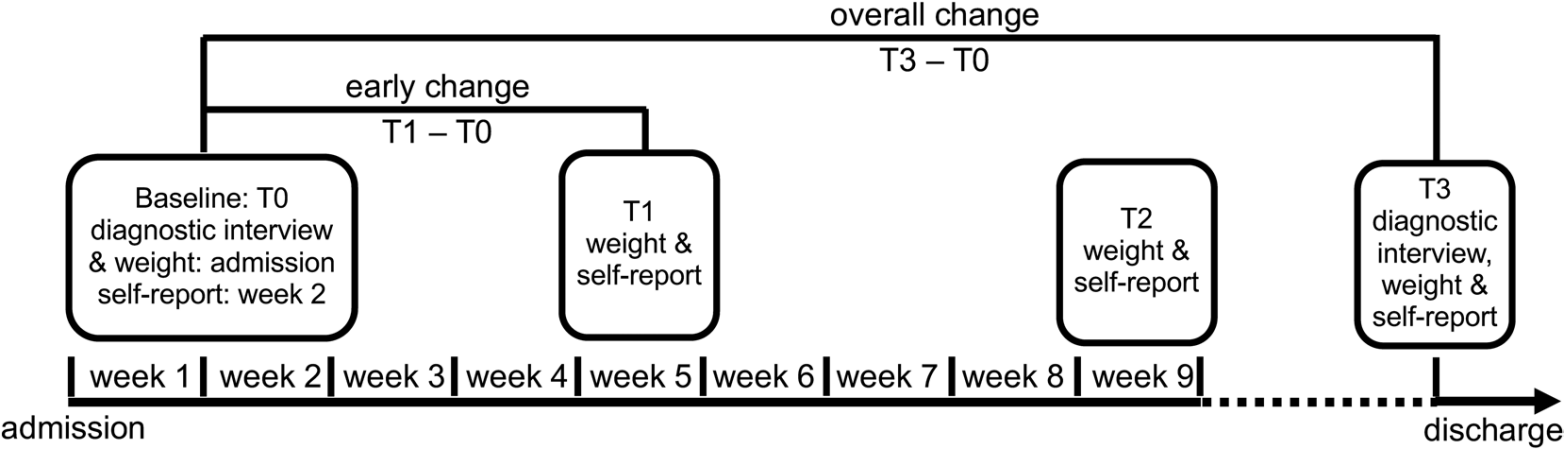
Procedure: time points of measurement.

All files of patients registered for seeking in‐patient ED treatment at Schoen Clinic Roseneck during the study period (11/2021–01/2023) were screened for study criteria. For adolescents, parents received study information and provided written informed consent. All positively screened patients were given study information in a personal appointment shortly after admission; written informed consent was obtained. Subsequently, the clinical interview was carried out and repeated at the last assessment. To facilitate that all patients had at least one regular therapy session with their individual therapist (after the admission session) baseline for the self‐report questionnaires was set to week 2 (7–14 days after admission). They were administered again during the fifth and ninth week of treatment as well as shortly before discharge (Figure 1). Ethical approval was granted by the ethical committee of Freiburg University. This study was preregistered on 25/08/2021 using the open science platform OSF (Werz 2021).

### 2.3 Measures

#### 2.3.1 AN Diagnosis

AN diagnosis was assessed in an interview with a clinical expert for eating disorders. The structured “International Diagnostic Checklists for ICD‐10 (IDCL)” (Hiller et al. 1997) were used, omitting criterion D of Anorexia nervosa (endocrine disorder), in line with the adjustments made in DSM‐5 and ICD 11.

#### 2.3.2 Remission Status

Remission status was assessed at the end of treatment during another clinical interview using the IDCL. Primary criterion was defined as underweight; secondary criteria were body image disturbances and behavior aimed at weight reduction (AN).

Full remission was defined as the presence of no more than one secondary criterion; partial remission was defined as the presence of no more than one primary or two secondary criteria to reflect meaningful clinical improvement. This includes cases where patients, despite still meeting one core criterion, show substantial progress in cognitive and behavioral symptoms during inpatient treatment; nonremission was defined as the presence of more criteria.

#### 2.3.3 Premature Treatment Termination

Reason for discharge was assessed by self‐report, patient file, and in case of doubt by consulting the treating team. Premature treatment termination was defined as either patient‐initiated discharge despite a recommendation to continue treatment (*n* = 54) or team‐initiated discharge due to insufficient adherence, such as consistent refusal to maintain adequate meal sizes (*n* = 4). This will hereafter be referred to as “termination.” Patients (*n* = 10) who had to terminate treatment due to transference to either another hospital (e.g., somatic reasons or high suicidality) or the intermediate care unit for extremely underweight patients (BMI < 13 kg/m^2^) within the same hospital were not included in analyses regarding termination, as they did neither terminate treatment as described above nor complete treatment.

#### 2.3.4 ED Symptomatology

The Eating Disorder Examination Questionnaire (EDE‐Q: Fairburn and Beglin 1994; German version: Hilbert et al. 2007) is a well‐established 28‐item self‐report instrument assessing ED symptoms over the past 28 days, using a 0–6 Likert scale. To measure severity, we utilized the global EDE‐Q scale. Psychometric properties of the EDE‐Q are good to very good. Height was measured at admission and weight was measured 1–2×/week as part of the treatment protocol and was extracted from patient files to calculate BMI. Normal weight was defined as BMI ≥ 18.5 kg/m^2^ for both adults and adolescents (≥ 14 years), based on guideline thresholds suggesting comparable values for both age groups (APA 2023; Herpertz et al. 2019). Weight status at discharge was defined as binary, based on the threshold for normal weight.

#### 2.3.5 Therapeutic Alliance

The Working alliance inventory‐revised‐short form (WAI‐SR; Hatcher and Gillaspy 2006; German Version: Wilmers et al. 2008) is a 12‐item self‐report questionnaire. While the original WAI (Horvath and Greenberg 1989) used a Likert scale ranging from 1 to 7, Hatcher and Gillaspy (2006) recommended in their revision the use of a 5‐point Likert scale (1–5), which was implemented in the German version. As we used the recommended revised 5‐point scaling of the WAI, these values cannot be directly compared with studies using the 7‐point scale. The WAI‐SR is based on the concept of TA by Bordin (1979), containing three subscales: shared goals, shared tasks, and emotional bond. The subscales and the global scale demonstrate good psychometric properties across studies (Hatcher and Gillaspy 2006; Wilmers et al. 2008). Patients were advised to refer to the relationship with their individual therapist for the WAI.

#### 2.3.6 Sociodemographic and Disorder‐Related Data

Standard sociodemographic questions (e.g., age, gender) were assessed. Participants gave information on the duration of illness, number and duration of previous inpatient treatments, and number of therapists. Comorbidities were retrieved from patient files.

#### 2.3.7 Motivation of Change

Motivation of change was assessed, in preparation for further exploratory mediation analyses, which are not part of this report and will therefore not be discussed further.

### 2.4 Treatment

The voluntary inpatient treatment corresponds to the German S3 guidelines for the treatment of ED (Herpertz et al. 2019; Resmark et al. 2019). The multimodal CBT‐oriented treatment is carried out by a multidisciplinary team of trained clinical psychologists, psychiatrists, nurses, social workers, occupational, physio‐ and exercise therapists, and nutritionists. It includes individual and group therapy, supervised meals, ED‐symptom protocols, and clinical management of medical issues. While individual therapy is not manualized, it addresses topics based on ED‐specific treatment programs (e.g., Fairburn 2008). Additional therapies include sports and art therapy, social skills training, meal preparation classes, body image exposure, and disorder‐specific group therapy. Patients with comorbidities receive specific therapeutic elements (e.g., exposure‐based therapies for anxiety and trauma‐related disorders). The involvement of families is encouraged for all age groups; for adolescents, at least three joint family sessions are typically conducted. For underweight AN patients, the goal is a weight gain of 0.7–1.0 kg per week, achieved through a high‐calorie refeeding schedule implemented from admission day. It consists of three main meals (approximately 700 kcal each) per day and is adjusted if patients fail to achieve the expected weight gain. Patients do not receive nasogastric feeding, as normalization of eating behavior is one of the therapeutic goals.

### 2.5 Analysis

**FIGURE 2:**
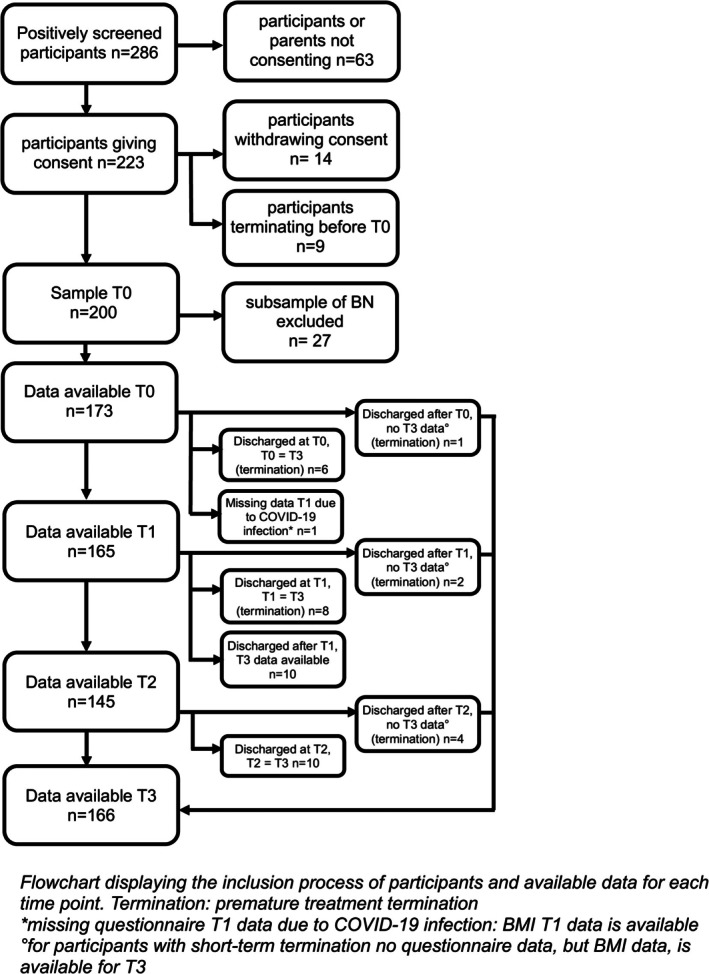
Flowchart.

Group differences of descriptive variables were calculated using t‐tests or Chi^2^‐tests (with exact Fisher test applied for the gender, due to low expected cell frequencies). There were no single missing data points (e.g., single items or questionnaires missing). However, not every patient participated at every time point, primarily due to treatment termination prior to week 5 or week 9 (see Figure 2). If week 2, 5, or 9 coincided with the week before discharge, data was used for both time points (*n* = 24). For some patients with short‐term termination, data collection before discharge was not possible (*n* = 7); however, data for BMI and weight status at discharge was available for all patients through their files. T0 refers to admission regarding weight measurement and to week 2 regarding self‐report questionnaires, T1 to week 5, T2 to week 9, and T3 to the week before discharge. Participants were included in the analyses for which data was available (T0: *n* = 173; T1: *n* = 165; T2: *n* = 145; T3: *n* = 166).

**FIGURE 3:**
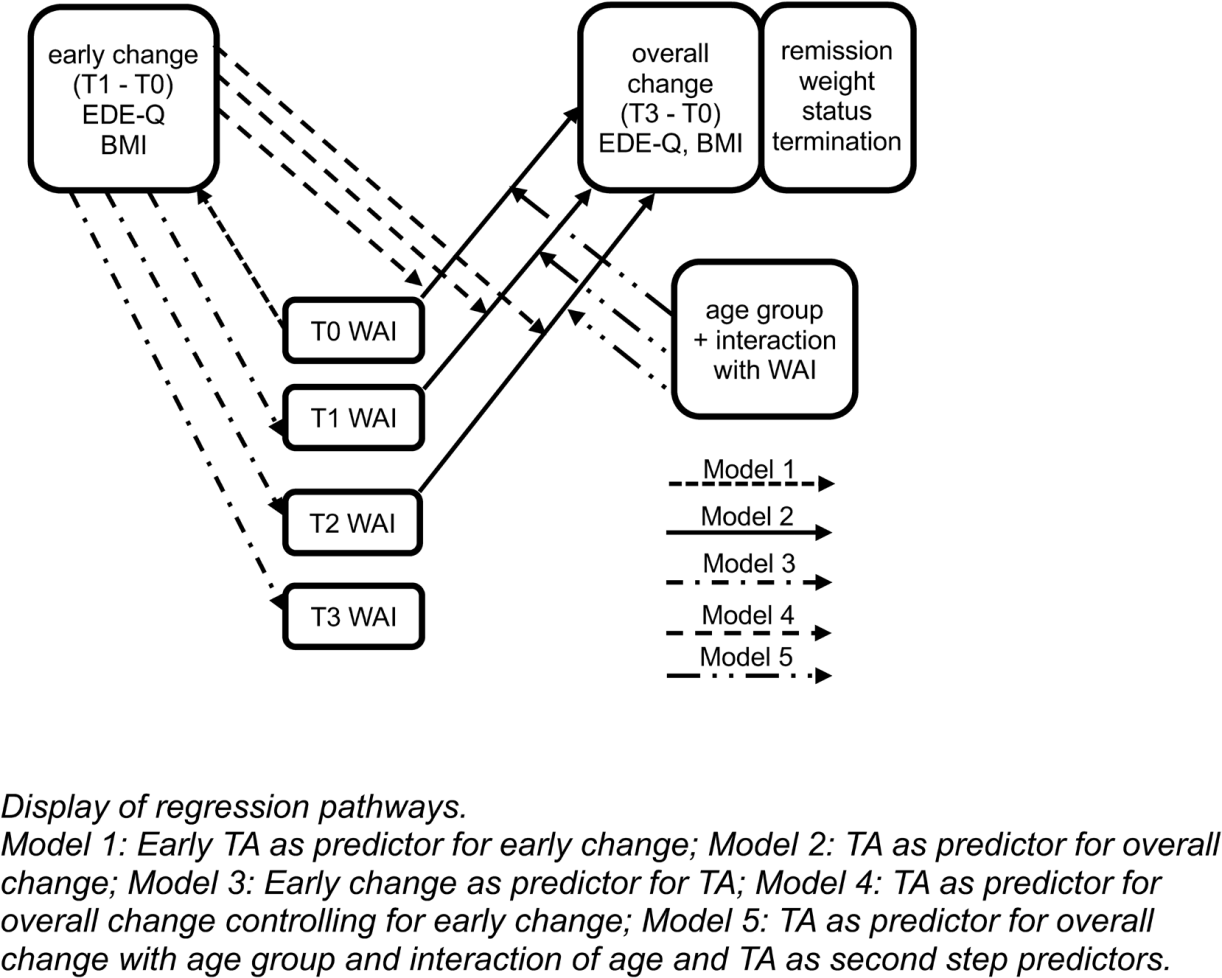
Regression pathways.

Several sets of multiple linear regressions (MLR), ordinal and binary logistic regressions were conducted (see Figure 3). Early change was defined as the difference between T0 and T1. Treatment outcome or overall change was defined as the difference between T0 and T3. For all difference scores, a positive value indicates positive change. All continuous variables were *z*‐standardized prior to analysis to facilitate comparability of regression coefficients across predictors and analyses. All analyses included the following variables as covariates: criterion variable at T0, duration of illness (DOI), gender, comorbidities, and prior treatment. Duration of treatment was included, except for the analyses concerning termination and early change. Prior treatment was measured with three variables (number and duration of previous inpatient treatments; number of previous therapists). For each analysis, it was determined which of these variables had the greatest impact on the criterion used, and final analyses were conducted with only this variable to prevent multicollinearity. While we used multiple comparisons, we tested models that were theory‐driven, aligned with primary study goals, and based on our hypotheses, determined before data collection and published in the study protocol. For all analyses, the respective statistical assumptions were tested for and sufficiently met or corrected for in case of violation. For analyses where TA showed to be a significant predictor, the analysis was repeated with the three subscales of the WAI to examine their specific effect.

Regressions were carried out in multiple series, according to our hypotheses.

For testing the prediction of outcome by TA:

(1) MLR with early TA as predictor for EDE‐Q/BMI (T0‐T1) as criterion.

(2a) MLR with TA at T0, T1, and T2 respectively as predictors for EDE‐Q/BMI (T0‐T3) as criterion.

(2b) Ordinal logistic regression with TA at T0, T1, and T2 respectively as predictors for remission status as criterion.

(2c) Binary logistic regressions with TA at T0, T1, and T2 respectively as predictors for termination and weight status as criteria.

For testing the prediction of TA by early change:

(3) MLRs with EDE‐Q and BMI (T0–T1) as predictor respectively for TA at T1, T2, and T3 as criteria.

For testing the prediction of outcome by TA, controlling for early change:

(4a–c) Regression analyses 2a–c, with the addition of respective early change variable as covariate.

For testing the effect of age and diagnosis on the relationship between TA and outcome:

For these analyses, it was first checked which timepoint (Tx) had the biggest effect in regression series 2, and only this TA was used:

(5a) Hierarchical MLR with EDE‐Q/BMI (T0‐T3) as criterion, TA at Tx and age group as the 1st step predictor, adding the interaction of TA and age group in the 2nd step.

(5b) Ordinal logistic regression with remission status as criterion, TA at Tx, and age group as the 1^st^ step predictor, adding the interaction of TA and age group in the 2nd step.

(5c) Binary logistic regressions with termination and weight status as criteria TA at Tx and age group as the 1st step predictor, adding the interaction of TA and age group in the 2nd step.

A priori power analyses were conducted using G*power. The model used was linear regression as described in Model 2a, with three predictors (TA at T0, T1, and T2) as this was our central hypothesis. The effect size Cohen's *f* ^2^ was used and, in accordance with previous research, conservatively estimated at 0.075, which lies between a small (0.02) and medium (0.15) effect according to established guidelines (Cohen 2013). The recommended minimum sample size was estimated to be *n* = 149. To ensure sufficient power and to obtain sufficiently large subsamples, we set the targeted sample size to *n* = 200. After achieving this sample size, we decided to exclude the small subsample of patients with BN (*n* = 27), as this subgroup was underpowered for analysis.

## 3 Results

### 3.1 Descriptive

Table 1 presents the descriptive data for the whole sample as well as the subsamples.

**TABLE 1:** Sample characteristics.

	Overall sample		Adults	Adolescents	Group differences (*t*‐test/Chi^2^ test) adults vs. adolescents		
	173		96	77			
*n*	M (SD)	Range	M (SD)	M (SD)	*t*	*p*	*d*
Age	20.77 (8.9)	14–63	24.92 (10.18)	15.61 (0.99)	8.9	**< 0.001**	1.22
No. comorbidities	1.31 (1.08)	0–5	1.57 (1.13)	0.97 (0.9)	3.87	**< 0.001**	0.58
Duration of treatment (weeks)	15.62 (7.11)	1.29–39	15.15 (7.13)	16.2 (7.08)	−0.97	NS	—
Prev. therapists (no.)	2.61 (2.34)	0–15	2.93 (2.82)	2.21 (1.47)	2.16	**0.016**	0.31
Prev. inpatient treatments (no.)	1.57 (1.98)	0–11	1.8 (2.32)	1.27 (1.43)	1.84	**0.033**	0.27

	M (SD)	Md	Range	M (SD)	Md	M (SD)	Md			
Prev. inpatient treatments (months)	7.59 (14.33)	3	0–120	10.35 (18.08)	4	4.14 (5.88)	2	3.17	**<0.001**	0.44
Duration of illness (months)	55.4 (79.63)	27	3–590	81.93 (98.2)	53.3	22.32 (17.3)	17	5.84	**<0.001**	0.8

	%	*n*	%	%	Chi^2^	*p*	φ
Gender					3.48	NS	—
Female	95.4	165	92.7	98.7			
Male	4.6	8	7.3	1.3			
Diagnosis					—	—	—
F50.00	110	63.6	61.5	66.2			
F50.01	49	28.3	30.2	26.0			
F50.08	1	0.6	0	1.3			
F50.1	13	7.5	8.3	6.5			
Remission at EoT					10.27	**0.006**	0.244
Full	30.1	52	39.6	18.2			
Part	32.9	57	31.3	35.1			
Non‐remission	37	64	29.2	46.8			
Termination^a^	39.3	68	31.3	49.4	5.87	**0.019**	−0.184

*Note*: The bold values are the significance values (*p*‐value).

Abbreviations: EoT: end of treatment; no: number; prev: previous.

^a^ Termination includes premature patient‐ or team‐initiated discharge and transference to another ward/hospital.

### 3.2 Change Over Time

Table 2 presents the change in symptoms and TA over the course of treatment. There was a significant improvement across all outcome variables with large effect sizes (partial eta^2^ > 0.4) (Cohen 2013). For general ED symptomatology as well as BMI, the change occurred during the whole course of treatment. TA improved steadily over the course of treatment. There were no significant group differences between adult and adolescent patients regarding baseline symptoms and baseline TA.

**TABLE 2:** TA and outcome over the course of treatment.

Measurement point										Repeated measures ANOVA			
	*N*	T0: week 2		T1: week 5		T2: week 9		T3: EoT		Greenhouse–Geisser			
		M	(SD)	M	(SD)	M	(SD)	M	(SD)	*F*	*p*	Multiple comparison (LSD—*p* < 0.001)	Partial eta^2^
WAI	141	3.32	(0.75)	3.53	(0.79)	3.74	(0.73)	3.93	(0.74)	46.6 (2.57, 360.08)	< 0.001	T0 < T1 < T2 < T3	0.421
EDEQ_Global	141	3.82	(1.35)	2.85	(1.35)	2.64	(1.36)	2.19	(1.38)	146.6 (2.36, 330.04)	< 0.001	T0 > T1 > T2 > T3	0.671
BMI	146	15.97	(1.86)	16.84	(1.72)	17.69	(1.61)	18.73	(1.52)	348.56 (1.56, 225.55)	< 0.001	T0 < T1 < T2 < T3	0.706

*Note*: The applied version of the WAI used a 5‐point Likertscale (1–5). Analyses were conducted for patients with data for all measurement points. BMI was available for 146 patients, EDEQ_Global and WAI for 141.

Abbreviations: BMI: body mass index; EDE‐Q: Eating Disorder Questionnaire; WAI: Working Alliance Questionnaire.

### 3.3 Regressions

Table 3 summarizes the results of MLR, limited to significant effects of the respective predictor variable(s) in question to enhance clarity. Hence, analyses that only showed significant effects of variables controlled for or already tested in a previous regression analysis, such as basic level of ED symptom at admission, are not listed but are available via Supporting Information (Supporting Table 1). Table 4 similarly presents the results of logistic regressions accordingly (Supporting Table 2).

**TABLE 3:** Multiple linear regression analyses.

Overall model						Predictors				
Criterion model (*n*)	Predictor examined	Overall *F*	(df)	*p*	*R* ^2^/adj. *R* ^2^	Significant predictors	Beta	*p*	Semipartial correlation	Explained variance
EC_BMI1 (*n* = 166)	WAI T0	4.597	(6159)	< 0.001	0.148/0.116	T0 BMI **T0 WAI**	−0.347 0.167	0.001 0.028	−0.338 0.162	11.4% 2.6%
	WAI‐Goal T0 WAI‐Task T0 WAI‐Bond T0	3.856	(6157)	< 0.001	1.64/1.22	T0 BMI **T0 WAI‐Task**	−0.332 0.207	< 0.001 0.036	−0.321 0.154	20.3% 2.4%
OC_EDEQ 2a (*n* = 142)	WAI T2	5.0	(7134)	< 0.001	0.207/0.166	T0 EDEQ **T2 WAI**	0.453 0.160	< 0.001 0.040	0.418 0.159	17.5% 2.5%
	WAI‐Goal T2 WAI‐Task T2 WAI‐Bond T2	4.387	(9132)	< 0.001	0.23/0.178	T0 EDEQ **T2 WAI‐Task**	0.459 0.279	< 0.001 0.016	0.424 0.186	18.0% 3.5%
WAI T23 (*n* = 145)	EC_EDEQ	13.73	(6138)	< 0.001	0.374/0.347	T0 WAI **EC_EDEQ**	0.563 0.205	< 0.001 0.003	0.555 0.203	30.8% 4.1%
WAI T23 (*n* = 146)	EC_BMI	12.878	(6139)	< 0.001	0.357/0.33	T0 WAI **EC_BMI**	0.562 0.162	< 0.001 0.021	0.554 0.159	30.7% 2.5%
OC_EDEQ 4a (*n* = 141)	WAI T2 EC_EDEQ	11.69	(8132)	< 0.001	0.415/0.379	T0 EDEQ **EC_EDEQ**	0.269 0.498	< 0.001 < 0.001	0.23 0.444	5.3% 19.7%
	WAI‐Goal T2 WAI‐Task T2 WAI‐Bond T2 EC_EDEQ	9.92	(10,130)	< 0.001	0.433/0.389	T0 EDEQ **EC_EDEQ** **T2 WAI‐Task**	0.279 0.49 0.206	< 0.001 < 0.001 0.041	0.237 0.436 0.136	5.6% 19.0% 1.9%
OC_BMI5a (*n* = 146)	WAI T2 Age group	18.109	(8137)	< 0.001	0.514/0.486	T0 BMIDOS **Age group**	−0.623 0.22 0.26	< 0.001 < 0.001 < 0.001	−0.61 0.208 0.231	37.2% 4.3% 5.3%

*Note*: Only regression analysis with significant examined predictors reported (for all results see Supporting Table 1). The bold entries significance values are provided in coumn *p*.

Abbreviations: BMI: body mass index; DOS: duration of stay; EC: early change; EDE‐Q: Eating Disorder Questionnaire; OC: overall change; PT: previous therapy; WAI: Working Alliance Questionnaire Global.

**TABLE 4:** Ordinal and binary logistic regression analyses.

Ordinal		Model fit			Predictors					
Criterion model (*n*)	Predictor examined	Chi^2^ (df)	*p*	Pseudo *R* ^2^ Nagelkerke	Significant predictors	B	Wald	*p*	OR	95% CI
Remission2b (*n* = 165)	WAI T1	19.29 (7)	0.007	0.124	DOS **T1 WAI**	0.372 0.32	5.002 4.34	0.025 0.037	1.451 1.377	1.047–2.010 1.019–1.861
Remission2b (*n* = 166)	WAI T2	15.16 (7)	0.034	0.111	**T2 WAI**	0.38	5.417	0.02	1.462	1.062–2.012
	WAI‐Goal T2 WAI‐Task T2 WAI‐Bond T2	19.23 (9)	0.023	0.139	**T2 WAI‐Task**	0.458	3.742	0.053	1.581	0.994–2.514
Remission 4b (*n* = 165)	WAI T1 EC_EDEQ	34.22 (8)	< 0.001	0.211	T0 EDEQ Comorbidity PT **EC_EDEQ**	−0.511 0.328 0.434 0.622	8.447 3.805 6.4 12.379	0.004 0.051 0.011 < 0.001	0.6 1.388 1.544 1.862	0.425–0.847 0.998–1.929 1.103–2.161 1.335–2.598
Remission 4b (*n* = 145)	WAI T2 EC_EDEQ	26.59 (8)	< 0.001	0.189	T0 EDEQ Comorbidity **EC_EDEQ**	−0.492 0.352 0.597	6.827 3.973 10.738	0.009 0.046 0.001	0.612 1.422 1.816	0.423–0.884 1.006–2.011 1.271–2.596
Remission 5b (*n* = 146)	WAI T2 Age group	23.4 (8)	0.003	0.167	**T2 WAI** **Age group**	0.351 −1.042	4.697 8.248	0.03 0.004	1.421 0.353	1.034–1.952 0.173–0.718

Binary		Model fit			Predictors					
Criterion model (*n*)	Predictor examined	Chi^2^ (df)	p	Pseudo *R* ^2^ Nagelkerke	Significant predictors	B	Wald	p	OR	95% CI
Weight status 5c (*n* = 146)	WAI T2 Age group	66.373 (8)	< 0.001	0.493	BMI T0 Duration of stay **Age group**	1.752 0.675 1.791	27.107 6.529 12.275	< 0.001 0.011 < 0.001	5.768 1.964 5.994	2.982–11.157 1.17–3.296 2.201–16.323
Termination 2c (*n* = 142)	WAI T2	17.79 (6)	0.007	0.168	Comorbidity **T2 WAI**	−0.531 −0.486	4.645 6.345	0.031 0.012	0.588 0.615	0.363–0.953 0.422–0.898
termination 4c (*n* = 141)	WAI T2 EC_EDEQ	18.48 (7)	0.01	0.176	Comorbidity **T2 WAI**	−0.554 −0.439	4.984 4.903	0.026 0.027	0.574 0.645	0.353–0.935 0.437–0.951
Termination 5c (*n* = 142)	WAI T2 Age group	24.15 (7)	< 0.001	0.224	**T2 WAI** **Age group**	−0.501 −1.149	6.409 6.022	0.011 0.014	0.606 0.317	0.411–0.893 0.127–0.794

*Note*: The groups were coded as following: remission: 0 = no remission, 1 = partial remission, 2 = full remission; weight status: 0 = BMI < 18.5 kg/m^2^, 1 = BMI ≥ 18.5 kg/m^2^; termination: 0 = no termination, 1 = termination; age group: −1 = adolescent, 1 = adult. Only regression analysis with significant examined predictors reported (for all results see Supporting Table 2). The bold entries significance values are provided in coumn *p*.

Abbreviations: BMI: body mass index; DOI: duration of illness; DOS: duration of stay; EC: early change; EDE‐Q: Eating Disorder Questionnaire; IA: interaction; OC: overall change; PT: previous therapy; WAI: Working Alliance Questionnaire.

#### 3.3.1 Influence of TA on Dimensional Outcome

TA at week 2 was a significant predictor for early change of BMI with 2.6% explained variance, but not for general ED symptoms. Looking into the subscales of TA, WAI‐Task was the significant subscale, while WAI‐Goal and WAI‐Bond had no significant effects. For overall change, TA at week 9 was a significant predictor of ED symptoms, with the subscale “task” explaining 3.5% variance. This effect was significant, but smaller when controlling for early change of ED symptoms, though early change had a large effect with 19.0% explained variance. There was no effect on overall change of BMI. Age group had a significant effect on overall change of BMI—but not general ED symptoms—with 5.3% explained variance and better outcome for adults. No interaction effect of age group with TA was significant.

#### 3.3.2 Influence of Early Change on TA

Early change of general ED symptoms (EDE‐Q) and BMI showed to be significant predictors for TA at week 9, with explained variances of 4.1% and 2.5%, respectively. Early change had no significant effect on TA at week 5 or before discharge.

#### 3.3.3 Influence of TA on Remission Status

TA at week 5 and at week 9 had a significant predictive effect on remission status, with effect sizes comparable to other variables like comorbidity and duration of stay (see Table 4). However, when controlling for early change of EDE‐Q, this effect ceased to be significant, while early change of EDE‐Q had a significant effect on remission. For TA at week 9, the subscale WAI‐Task showed a significant effect, while no single subscale had a significant effect for TA at week 5. There was no significant interaction effect of TA and age group, while age group was a significant predictor of remission status with an almost tripled probability for a better outcome in adults compared to adolescents.

#### 3.3.4 Influence of TA on Weight Status

There was no significant effect of TA at any timepoint on weight status at discharge. Early change of BMI and age group were significant predictors, with a sixfold higher probability of gaining normal weight for adults over adolescents. There was no interaction effect of age group and TA.

#### 3.3.5 Influence of TA on Premature Treatment Termination

There was no effect of TA at week 2 and week 5 on termination. TA at week 9 had a significant effect on termination, with a 1.5 higher probability of completing treatment regularly with one SD more of TA. This effect remained significant after controlling for early change of EDE‐Q, while early change had no significant effect. The analysis of TA subscales revealed no single significant subscale effect. No interaction effect between TA and age group was shown, while age group was a significant predictor with a three times higher probability of termination for adolescents.

## 4 Discussion

This study examined the relationship between TA, early symptom change, and outcome in a naturalistic sample of in‐patients suffering from AN being treated with ED‐specific CBT. Treatment was effective, with large effect sizes for the reduction of ED‐symptomatology over time. Simultaneously, TA increased over time, consistent with prior research (Brown et al. 2013a; Raykos et al. 2014; Stiles‐Shields et al. 2013).

### 4.1 Predictive Effect of Early TA on Early Change

Early TA did not predict early change of general ED symptoms, but did predict early change of BMI. As weight gain is a central goal in therapy but much feared by patients (Crone et al. 2023; Herpertz et al. 2019; Vinchenzo et al. 2022), even a small effect of 2.6% explained variance can be of significance for clinical work. The “first impression” during the first two sessions of therapy could positively impact this early treatment phase, potentially facilitating a positive trajectory for the therapeutic work. It is important to note that this effect is based on the TA subscale “task,” so facilitating an early agreement on what is necessary to be done during therapy is the crucial factor. However, it is important to note that given the small effect size, this finding should be interpreted with caution and not overestimated.

### 4.2 Predictive Effect of TA on Termination and Outcome at Discharge and Role of Early Change

TA later during treatment (week 9) did predict symptom improvement between admission and discharge for general ED symptoms, again with the task subscale as the crucial factor. While this effect is small, especially in comparison with the large effect of early change, it shows that the constant maintenance of an agreement on the therapeutic tasks should be kept in mind during the whole therapeutic process.

Accordingly, while earlier TA (week 2 and 5) did not predict termination, TA at week 9 remained a stable predictor for termination even after controlling for early change. This latter finding is in accordance with previous research (Jordan et al. 2017; Sly et al. 2013, 2014). Interestingly, neither baseline symptom severity nor early change did predict termination. Hence, this could be the factor where TA plays an important role over and above symptom reduction. Notably, this effect was not attributable to one specific subscale of TA.

The remission status was predicted by TA at weeks 5 and 9. A good TA may enhance the influence of therapists on their patients' decision regarding duration of stay. Considering the positive effect of duration of stay, there could be a mediating effect on remission status via duration of stay. However, the effect of TA vanished when controlling for early change of general ED symptoms, while early change predicted remission. Additionally, there was no prediction of weight status by TA. These results highlight the complexity of the relationship between early change, long‐term outcome, and TA over time, depending on confounding variables as well as the operationalization of outcome.

Early change of BMI and overall ED symptoms predicted TA at week 9, indicating a delayed positive effect of improvement during the first weeks of treatment on TA later in the treatment process. This result, emphasizing the bidirectional relationship of outcome and TA, corresponds with the findings of Graves et al. (2017) in their meta‐analysis. Additionally, early change was shown to have large predictive effects on all ED outcome variables, which is in line with a recent meta‐analysis showing early change to be a robust predictor of outcome in ED treatment (Chang et al. 2021).

Summing up, we found some support for our first and fourth hypotheses on TA's influence on ED symptoms, with the TA subscale task being most important. There also was some evidence for our second hypothesis regarding a prediction of termination by TA later in the therapeutic process. Our third hypothesis concerning the influence of early change on TA was only supported for later TA (week 9) by the results. TA seems to have small effects over and above the impact of early change on ED symptomatology over the entire duration of inpatient treatment, its most crucial role being the prevention of premature treatment termination.

### 4.3 Predictive Effect of Age

In contrast to our fifth hypothesis, the effect of TA did not interact with age group. Age was an important factor, with a distinctly better outcome for BMI and lower termination rates for adults, although adults had significantly more comorbidities, previous therapy, and a longer duration of illness—variables that are associated with a negative impact on outcome (Bandini et al. 2006; Fernández‐Aranda et al. 2021; Gorrell et al. 2023). There are different possible explanations for those results: Intrinsic motivation to change, which poses as a positive predictor for outcome (Carter and Kelly 2015) may be lower in adolescents, who often seek treatment due to their parents and not self‐motivated. Most studies examining age as an influencing factor concentrate on age of onset, and few studies compare treatment outcome for both age groups treated in a comparable setting. Calugi et al. (2015) found better outcome for adolescents, with more young patients reaching goal BMI and faster weight gain in an outpatient setting. Adolescents may suffer from homesickness and the separation from friends and families in an inpatient setting more than adults, leading them to leave treatment as soon as possible (partial remission). Hence, day clinics and new treatment approaches like outreach home treatment may be more suitable for these patients (Herpertz‐Dahlmann et al. 2021).

### 4.4 Strengths and Limitations

Our study had some limitations: Due to a small subsample of BN patients, resulting in an underpowered BN subsample, we had to exclude BN patients from our analyses. We only determined the full sample size in our power analyses without setting a minimum subsample size, which we would strongly recommend for future studies.

We analyzed our data using a between‐patient design, comparing patients regarding their TA and outcome. However, recent research (Flückiger et al. 2020) indicates that a within‐patient design, looking into the relationship of TA and symptoms in each patient on a session‐to‐session basis, could be an important perspective. While we assessed TA more often than many studies (Graves et al. 2017; Werz et al. 2022), four assessments over an average inpatient stay of 15 weeks still only allow for a rough picture. As the relationships between variables in the therapeutic process appear to relate in a complex way, this frequency may still be too limited to give a full picture.

Additionally, the small effects of TA should be interpreted cautiously in the context of a multimodal in‐patient treatment, which includes numerous therapeutic modalities, multiple therapists, and close interactions with other patients. While the individual therapist serves as the primary therapeutic facilitator—responsible for planning other treatment components and engaging with patients during group sessions and meals—the assessed individual therapy is still only one element among many; therefore, its specific impact may be overshadowed by the multiple concurrent influences inherent to the inpatient environment and may not be directly comparable to an outpatient setting with only individual therapy.

There are several distinctive strengths in our study. We used a naturalistic sample containing patients of different age groups treated in the same setting. The severity of illness, as reflected in baseline ED symptoms, was substantial in our sample. In contrast to most previous studies examining TA in ED, we conducted an a priori power analysis and had a rather large sample size. Our study examined TA as a primary aim and used theory‐based hypotheses rather than exploratory secondary analyses. We assessed TA early in the treatment course and repeatedly during the therapeutic process. The inclusion of multiple influencing variables like previous treatments and comorbidities improves the practical impact of our results. This is especially important for considering the effect of early change, as there seems to be a close interconnection between early change and TA.

### 4.5 Future Research

We emphasize the need for further studies using a fine‐grained, ideally session‐to‐session assessment of TA and symptomatology. Analyses should consider within‐patient effects and investigate further variables that could serve as a mediator/moderator for the effect of TA on outcome, such as motivation to change or honesty in therapy. Examining the influence of therapist‐related and setting factors, such as experience, therapeutic style, and inpatient vs. outpatient treatment could shed more light on these complex relationships.

### 4.6 Conclusion and Clinical Implications

Our study showed that the effect of TA on ED symptoms through the course of treatment is less pronounced than the effect on other disorders—however, the effect of the inpatient setting may overemphasize this difference. Established TA after some weeks of treatment may pose as a protection factor regarding premature treatment termination. In line with previous research, the improvement in symptoms during the first weeks of treatment seems to be an important and robust factor, not only positively influencing outcome but also the maintenance of a good TA later in therapy. Early change can be enhanced by a good first impression reflected in early TA. The most relevant part of TA seems to be the agreement on therapeutic tasks. These results have strong clinical implications and indicate the importance of a symptom‐focused, directive therapeutic stance. The fear of compromising TA with a clear stance, encouraging quick changes in eating behavior and promoting weight gain may not overwhelm patients. If there is a good TA basis, this attitude rather enhances TA further, via the facilitation of early change.

## Author Contributions

**Janina Werz:** conceptualization, investigation, writing – original draft, methodology, writing – review and editing, formal analysis, project administration, data curation. **Brunna Tuschen‐Caffier:** writing – review and editing, supervision. **Ulrich Voderholzer:** writing – review and editing, supervision, resources.

## Conflicts of Interest

The authors declare no conflicts of interest.

## Supporting information


**Data S1:** eat24524‐sup‐0001‐Tables.doc.

## Data Availability

The data that support the findings of this study are available from the corresponding author upon reasonable request.
