# Supplementary material for: The Relationship Between Therapeutic Alliance, Early Symptom Change, and Outcome Among In‐Patients With Anorexia Nervosa
Source: Int J Eat Disord. 2025 Sep 24;58(12):2378–91. doi: 10.1002/eat.24524 (PMC12703211; doi:10.1002/eat.24524)
Supplement: Supplementary file 1 — Data S1: eat24524‐sup‐0001‐Tables.doc. [file EAT-58-2378-s001.pdf]

Supplementary Table 1: Multiple linear regression analyses

Overall model						predictors				
Criterion Model (n)	predictor examined	Overall F	(df)	p	R^2/adj. R^2	significant predictors	beta	p	semipartial correlation	explained variance
EC_EDEQ						T0 EDEQ	.323	<.001	.313	9.8%
1 (n=165)	**WAI T0**	3.68	(6,158)	.002	.123 / .089	comorbidity	-.178	.028	-.166	2.8%
EC_BMI						**T0 WAI**	.167	.028	.162	2.6%
1 (n=166)	**WAI T0**	4.6	(6,159)	<.001	.148 /.116	T0 BMI	-.347	.001	-.338	11.4%
OC_EDEQ										
2a (n=166)	**WAI T0**	4.78	(7,158)	<.001	.175 / .138	T0 EDEQ	.380	<.001	.352	12.4%
OC_EDEQ										
2a (n = 159)	**WAI T1**	4.22	(7,151)	<.001	.164 / .125	T0 EDEQ	.394	<.001	.368	13.5%
OC_EDEQ						T0 EDEQ	.453	<.001	.418	17.5%
2a (n=142)	**WAI T2**	5.0	(7,134)	<.001	.207 / .166	**T2 WAI**	.160	.040	.159	2.5%
OC_BMI						T0 BMI	-.485	<.001	-.473	22.4%
2a n=173)	**WAI T0**	17.16	(7,165)	<.001	.421 / .397	DOS	.393	<.001	.374	14.0%
OC_BMI						T0 BMI	-.506	<.001	-.494	24.4%
2a (n=165)	**WAI T1**	15.4	(7,157)	<.001	.407 / .381	DOS	.338	<.001	.322	10.4%
OC_BMI						T0 BMI	-.616	<.001	-.603	36.4%
2a (n=146)	**WAI T2**	16.84	(7,138)	<.001	.461 / .433	DOS	.203	.003	.192	3.7%
WAI T1						T0 WAI	.614	<.001	.609	37.1%
3 (n=165)	**EC_EDEQ**	19.47	(6,158)	<.001	.435 / .403	DOI	.161	.014	.151	2.3%
WAI T2						T0 WAI	.563	<.001	.555	30.8%
3 (n=145)	**EC_EDEQ**	13.73	(6,138)	<.001	.374 / .347	**EC_EDEQ**	.205	.003	.203	4.1%
WAI T3										
3 (n=159)	**EC_EDEQ**	8.39	(6,152)	<.001	.249 / .219	T0 WAI	.473	<.001	.468	21.9%
WAI T1						T0 WAI	.606	<.001	.595	35.4%
3 (n=165)	**EC_BMI**	19.47	(6,158)	<.001	.425 / .403	DOI	.161	.014	.15	2.3%
WAI T2						T0 WAI	.562	<.001	.554	30.7%
3 (n=146)	**EC_BMI**	12.88	(6,139)	<.001	.357 / .33	**EC_BMI**	.162	.021	.159	2.5%
WAI T3										
3 (n=160)	**EC_BMI**	8.1	(6,153)	<.001	.24 / .21	T0 WAI	.47	<.001	.46	21.2%
OC_EDEQ	**WAI T0**					T0 EDEQ	.202	.005	.179	3.2%
4a (n=159)	**EC_EDEQ**	13.16	(8,150)	<.001	.412 / .381	**EC_EDEQ**	.535	<.001	.503	25.3%
OC_EDEQ	**WAI T1**					T0 EDEQ	.201	.005	.177	3.1%
4a (n=159)	**EC_EDEQ**	13.13	(8,150)	<.001	.412 / .381	**EC_EDEQ**	.537	<.001	.498	24.8%
OC_EDEQ	**WAI T2**					T0 EDEQ	.269	<.001	.23	5.3%
4a (n=141)	**EC_EDEQ**	11.69	(8,132)	<.001	.415 / .379	**EC_EDEQ**	.498	<.001	.444	19.7%
						T0 BMI	-.354	<.001	-.323	10.4%
OC_BMI	**WAI T0**					DOS	.435	<.001	.356	12.7%
4a (n=166)	**EC_BMI**	25.53	(8,157)	<.001	.565 / .543	**EC_BMI**	.435	<.001	.4	16.0%
						T0 BMI	-.343	<.001	-.312	9.7%
						DOS	.380	<.001	.361	13.0%
OC_BMI	**WAI T1**					PT	-.109	.054	-.102	16.2%
4a (n=165)	**EC_BMI**	25.82	(8,156)	<.001	.57 / .548	**EC_BMI**	.445	<.001	.403	16.2%
						T0 BMI	-.469	<.001	-.413	17.1%
OC_BMI	**WAI T2**					DOS	.26	<.001	.242	5.9%
4a (n=14a6)	**EC_BMI**	20.88	(8,137)	<.001	.549 / .523	**EC_BMI**	.344	<.001	.406	16.5%
OC_EDEQ	**WAI T2**					T0 EDEQ	.454	<.001	. 417	17.4%
5a (n=142)	**age group**	4.35	(8,133)	<.001	.207 / .159	**T2 WAI**	.159	.042	.159	2.5%
	**WAI T2**									
OC_EDEQ	**age group**					T0 EDEQ	.453	<.001	.413	17.1%
5a (n=142)	**IA WAI x age**	3.83	(9,132)	<.001	.207 / .153	**T2 WAI**	.159	.046	.156	2.4%

						T0 BMI	-.623	<.001	-.61	37.2%
OC_BMI	**WAI T2**					DOS	.22	<.001	.208	4.3%
5a (n=146)	**age group**	18.11	(8,137)	<.001	.514 / .486	**age group**	.26	<.001	.231	5.3%
	**WAI T2**					T0 BMI	-.627	<.001	-.606	36.7%
OC_BMI	**agegroup**					DOS	.247	<.001	.207	4.3%
5a (n=146)	**IA WAI x age**	16.02	(9,136)	<.001	.515 / .482	**age group**	.26	<.001	.231	5.4%

Notes: Only significant predictors reported. Abbreviations: BMI: Body mass index; DOI: duration of illness; DOS: duration of stay; EC: early change; EDE-Q: Eating Disorder Questionnaire; IA: interaction; OC: overall change; PT: previous therapy; WAI: Working Alliance Questionnaire.

Supplementary Table 2: Ordinal and binary logistic regression analyses

Ordinal		model fit			predictors					
Criterion Model (n)	predictor examined	Chi^2 (df)	p	Pseudo R^2 Nagelkerke	significant predictors	B	Wald	p	OR	95% CI
remission 2b (n=173)	**WAI T0**	23.85(7)	.001	.145	DOS	.518	10.302	.001	1.678	1.223 - 2.302
remission 2b (n=165)	**WAI T1**	19.29(7)	.007	.124	DOS	.372	5.002	.025	1.451	1.047 - 2.010
					**T1 WAI**	.32	4.34	.037	1.377	1.019 - 1.861
remission 2b (n=166)	**WAI T2**	15.16(7)	.034	.111	**T2 WAI**	.38	5.417	.02	1.462	1.062 - 2.012
					T0 EDEQ	-.531	9.22	.002	0.588	0.417 - 0.828
					comorbidity	.33	3.847	.05	1.39	1.000 - 1.933
remission 4b (n=165)	**WAI T0**				DOS	.452	6.909	.009	1.571	1.122 - 2.200
	**EC_EDEQ**	34.02(8)	<.001	.21	**EC_EDEQ**	.645	14.673	<.001	1.907	1-370 - 2.652
					T0 EDEQ	-.511	8.447	.004	0.6	0.425 - 0.847
					comorbidity	.328	3.805	.051	1.388	0.998 - 1.929
remission 4b (n=165)	**WAI T1**				PT	.434	6.4	.011	1.544	1.103 - 2.161
	**EC_EDEQ**	34.22(8)	<.001	.211	**EC_EDEQ**	.622	12.379	<.001	1.862	1.335 - 2.598
					T0 EDEQ	-.492	6.827	.009	0.612	0.423 - 0.884
remission 4b (n=145)	**WAI T2**				comorbidity	.352	3.973	.046	1.422	1.006 - 2.011
	**EC_EDEQ**	26.59(8)	<.001	.189	**EC_EDEQ**	.597	10.738	.001	1.816	1.271 - 2.596
remission 5b (n=146)	**WAI T2**				**T2 WAI**	.351	4.697	.03	1.421	1.034 - 1.952
	**age group**	23.4(8)	.003	.167	**age group**	-1.042	8.248	.004	0.353	0.173 - 0.718
	**WAI T2**									
remission 5b (n=146)	**age group**				**T2 WAI**	.376	4.944	.026	1.457	1.046 - 2.03
	**IA WAI x age**	24.2(9)	.004	.172	**age group**	-1.062	8.541	.003	0.346	0.17 - 0.705
Binary		model fit			predictors					
Criterion Model (n)	predictor examined	Chi^2 (df)	p	Pseudo R^2 Nagelkerke	significant predictors	B	Wald	p	OR	95% CI
weight status 2c (n=173)	**WAI T0**	74.98(7)	<.001	.47	T0 BMI	1.654	32.606	<.001	5.227	2.963 - 9.222
					DOS	0.955	17.022	<.001	2.598	1.651 - 4.089
weight status 2c (n=165)	**WAI T1**	68.85(7)	<.001	.456	T0 BMI	1.669	32.075	<.001	5.306	2.978 - 9.455
					DOS	0.838	12.436	<.001	2.312	1.451 - 4.683
weight status 2c (n=146)	**WAI T2**	52.46(7)	<.001	.408	T0 BMI	1.558	27.005	<.001	4.751	2.639 - 8.551
					DOS	0.612	5.651	.017	1.844	1.113 - 3.054
weight status 4c (n=166)	**WAI T0**	93.11(8)	<.001	.575	T0 BMI	2.424	37.513	<.001	11.29	5.198 - 24.52
					DOS	1.080	16.708	<.001	2.944	1.754 - 4.941
	**EC_BMI**				**EC_BMI**	1.331	18.601	<.001	3.785	2.067 - 6.930
weight status 4c (n=165)	**WAI T1**	91.76(8)	<.001	.571	T0 BMI	2.408	36.655	<.001	11.111	5.096 - 24.226
					DOS	1.07	16.464	<.001	2.916	1.739 - 4.890
	**EC_BMI**				**EC_BMI**	1.296	17.278	<.001	3.654	1.983 - 6.732
weight status 4c (n=146)	**WAI T2**	70.69(8)	<.001	.518	T0 BMI	2.27	31.594	<.001	9.675	4.385 - 21.35
					DOS	0.846	9.397	.002	2.329	1.357 - 4.000
	**EC_BMI**				**EC_BMI**	1.202	14.415	<.001	3.328	1.789 - 6.192
weight status 5c (n=146)	**WAI T2**	66.37(8)	<.001	.493	T0 BMI	1.752	27.107	<.001	5.768	2.982 - 11.16
					DOS	0.675	6.529	.011	1.964	1.170 - 3.296
	**age group**				**age group**	1.791	12.275	<.001	5.994	2.201 - 16.32
weight status 5c (n=146)	**WAI T2**	66.72(9)	<.001	.495	T0 BMI	1.732	26.258	<.001	5.652	2.914 - 10.96
	**age group**				DOS	0.668	6.372	.012	1.951	1.161 - 3.278
	**IA WAI x age**				**age group**	1.785	12.179	<.001	5.959	2.187 - 16.24
termination 2c (n=163)	**WAI T0**	13.44(6)	.037	.109	comorbidity	-.575	7.086	.008	0.563	0.369 - 0.859
termination 2c (n=156)	**WAI T1**	13.74(6)	.033	.118	comorbidity	-.555	6.232	.013	0.574	0.371 - 0.888
termination 2c (n=142)	**WAI T2**	17.79(6)	.007	.168	comorbidity	-.531	4.645	.031	0.588	0.363 - 0.953
					**T2 WAI**	-.486	6.345	.012	0.615	0.422 - 0.898

termination	**WAI T0**									
4c (n=156)	**EC_EDEQ**	14.05(7)	.050	.12	comorbidity	-.604	7.248	.007	0.546	0.352 - 0.848
termination	**WAI T1**									
4c (n=156)	**EC_EDEQ**	15.79(7)	.027	.134	comorbidity	-.597	7.02	.008	0.551	0.354 - 0.856
termination	**WAI T2**				comorbidity	-.554	4.984	.026	0.574	0.353 - 0.935
4c (n=141)	**EC_EDEQ**	18.48(7)	.01	.176	**T2 WAI**	-.439	4.903	.027	0.645	0.437 - 0.951
termination	**WAI T2**				**T2 WAI**	-.501	6.409	.011	0.606	0.411 - 0.893
5c (n=142)	**age group**	24.15(7)	<.001	.224	**age group**	-1.149	6.022	.014	0.317	0.127 - 0.794
	**WAI T2**									
termination	**age group**				**T2 WAI**	-.51	6.383	.012	0.6	0.404 - 0.892
5c (n=142)	**IA WAI x age**	24.32(8)	.002	.225	**age group**	-1.127	5.767	.016	0.324	0.129 - 0.813

Notes: The groups were coded as following: remission: 0 = no remission, 1= partial remission 2 = full remission; weight status: 0 = BMI < 18,5 kg/m^2, 1 = BMI >= 18,5 kg/m^2; termination: 0 = no termination, 1 = termination; age group: -1 = adolescent, 1= adult. Only significant predictors reported. Abbreviations: BMI: Body mass index; DOI: duration of illness; DOS: duration of stay; EC: early change; EDE-Q: Eating Disorder Questionnaire; IA: interaction; OC: overall change; PT: previous therapy; WAI: Working Alliance Questionnaire.
